# Associations between manure pumping out of the site and exposure to nearby manure applications with the incidence of porcine reproductive and respiratory syndrome virus and porcine epidemic diarrhea virus outbreaks in wean-to-market pig populations in the Midwestern United States

**DOI:** 10.3389/fvets.2025.1595313

**Published:** 2025-09-02

**Authors:** Ana Paula Serafini Poeta Silva, Cesar Amorim Moura, Nicholas Johansen, Daniel Moraes, Rafael Romero Nicolino, Edison Sousa Magalhães, Kinath Rupasinghe, Swaminathan Jayaraman, Christopher Rademacher, Giovani Trevisan, Daniel C. L. Linhares, Gustavo S. Silva

**Affiliations:** ^1^Veterinary Diagnostic and Production Animal Medicine, Iowa State University, Ames, IA, United States; ^2^Iowa Select Farms, Iowa Falls, IA, United States; ^3^Department of Preventive Veterinary Medicine, School of Veterinary Medicine, Federal University of Minas Gerais, Belo Horizonte, Minas Gerais, Brazil

**Keywords:** PRRSV, PEDV, biosecurity, manure, spread, pumping, nursery, grow-finish

## Abstract

**Introduction:**

Manure handling is an integral part of swine production, providing valuable nutrients for crop fields but also posing potential biosecurity risks. This study investigated the association between manure removal and nearby manure applications with the occurrence of porcine reproductive and respiratory syndrome virus (PRRSV) and porcine epidemic diarrhea virus (PEDV) outbreaks in wean-to-market pig lots.

**Methods:**

Data from a swine production system were analyzed for the period between July 2020 and December 2022 using a matched case–control design. Cases were defined as PRRSV or PEDV outbreaks confirmed through veterinary assessment and pathogen RNA detection. Manure exposure was assessed at the site level, including (1) pumping manure out from the site and (2) proximity (≤1.61, 4.82, and 8.04 km) to fields receiving manure. Conditional logistic regression models evaluated outbreak odds ratios within 5 weeks of exposure. Additional mixed effects models identified risk factors associated with PRRSV outbreaks.

**Results:**

Among 2,592 wean-to-market lots across 599 sites, 1,370 lots had at least one manure pumping event, 380 PRRSV outbreaks, and 103 PEDV outbreaks. PRRSV outbreaks were significantly associated (*p*-value < 0.05) with manure pumping (Odds Ratio [OR] = 3.38, 95% CI: 1.86–6.11) and proximity to fields receiving manure at distances of ≤1.61 km (OR = 4.09, 95% CI: 1.05–16.00) and 4.82 km (OR = 3.05, 95% CI: 1.12–8.27). Significant risk factors for PRRSV outbreaks after manure pumping included herd size (>10,000 pigs: OR = 6.75, 95% CI: 3.24–14.06), week of pumping (1st–4th week post-placement: OR = 5.64, 95% CI: 1.76–18.08), and prior PRRSV-positive lots (OR = 3.52, 95% CI: 1.55–7.97). PRRSV outbreaks following manure exposure from adjacent fields were more likely in large herds (>10,000 pigs: OR = 11.47, 95% CI: 3.43–44.52) and at closer distances <1.61 km (OR = 11.3, 95% CI: 2.73–53.43). No significant associations were found between PEDV outbreaks and manure exposure, likely due to the limited observations in this study.

**Discussion:**

These findings highlight the significance of manure management in PRRSV transmission risk, particularly the timing of manure pumping, herd size, and proximity to manure-applied fields. Improving biosecurity measures during manure handling can help lower PRRSV transmission risks in swine production.

## 1 Introduction

Livestock manure (or waste) is a byproduct of animal farming and an abundant source of macro and micronutrients, making it an efficient crop fertilizer (1, 2). The production of manure in swine farms is significant. For instance, in Iowa USA, swine farms produce over 50 × 10^6^ Mg of wet-basis manure yearly (3). The procedures for manure management typically involve storage, preparation for application (e.g., homogenization and pumping out of the storage facility), and spreading to crop fields. Manure produced in Midwestern United States pig wean-to-market facilities is usually stored in concrete deep pits 1.8–2.5 m beneath the building (4). Deep pits are common due to their capability of conserving nutrients for longer periods. Compared to other storage methods, deep pits can retain more nitrogen, phosphorus, and potassium in the manure (5). Other manure storage systems are also used in swine farms in the United States, including aboveground tanks, and open earthen lagoons (5).

Manure is pumped from facility structures (e.g., a deep pit under the animal housing facility or a lagoon attached to the site) on an annual or semi-annual basis due to the limited storage capacity of the pits and earthen lagoons, but also to align with crop fertilization schedules before the planting season. For instance, in the Midwestern United States, manure is typically applied in the fall after harvest or in early spring prior to planting, depending on weather conditions, soil saturation, and nutrient management plans. Because pigs produce manure continuously, storage pits gradually fill and must be emptied periodically to prevent overflow and maintain facility function.

Following manure homogenization and agitation (namely pumping) in the deep pits or lagoons, the manure is transferred to a tank hauled by a truck or directly to the drag hose (with a maximum length of 3.22 km [2 miles]) spreader for transportation to the application sites (6). The collected manure is then spread onto crop fields using specialized equipment, such as broadcast spreading (manure is distributed evenly over the soil surface), banded application (manure is applied in narrow bands or strips on the soil), and direct injection (manure is injected directly into the soil, reducing surface exposure) (7). While these practices are essential for nutrient recycling and waste management, they may also play a role in pathogen dissemination within and between swine production sites. That is, manure pumping and spreading in the crops can generate airborne particles and move infectious pathogens from site to site. Pu et al. (8) showed that manure handling, including storage and pumping in wean-to-finish facilities, produced airborne particles of 2.5 micrometers or smaller in diameter, which can be inhaled into the lungs of pigs and pig producers.

Porcine reproductive and respiratory syndrome virus (PRRSV) and porcine epidemic diarrhea virus (PEDV) are major economic and pig welfare threats to wean-to-market pig populations, associated with increased mortality rates in weaning and growing pig populations (9–11). The Swine Disease Reporting System, a USA-based veterinary disease surveillance program designed to monitor major swine pathogens, has consistently reported PRRSV RNA detection rates ranging from 35 to 45% in wean-to-market pig populations since 2021, depending on the season (12). In contrast, PEDV RNA detection during the same period has ranged from 10 to 20% seasonally (12). As described elsewhere (13–15), infected pig populations can shed these viruses for at least 4 weeks post-infection, enabling continued transmission to other herds via long-distance airborne particles (16, 17).

Studies in controlled and field settings revealed that PRRSV can be detected by PCR and isolated from air and fecal samples, enabling its potential indirect transmission among pig populations. Viable PRRSV was recovered from swine slurry, e.g., up to 28 days at 4 °C and 7 days at 20 °C (18), and pig manure, e.g., up to 35 days at 4 °C and 7 days at 25 °C (19). Furthermore, PRRSV was isolated and sequenced (ORF5 genes) from 120 PRRSV RNA-positive samples by PCR in China (20). PRRSV RNA was detected at 9.1 km from the infectious source (growing pigs) in the air, and following bioassay testing revealed that PRRSV RNA-positive air samples were infectious (10, 21, 22). Likewise, PEDV survived for at least 7 days in pig feces stored at room temperature and up to 28 days in feces stored at 4 °C (23). PEDV RNA was detected at 16.1 km downwind from the infectious source (pigs inoculated with PEDV) (17). More recently, Montoya et al. (24) reported that among 385 manure pit samples collected from pig farms in Minnesota and Iowa, 7.75% tested positive for PRRSV RNA and 13.79% for PEDV RNA. In another study, Moraes et al. (25) collected four manure pit samples from each of 75 wean-to-finish farms in Iowa during the two-week period before and after manure pumping. They observed that the detection rate of PRRSV RNA in manure pit samples increased from 16.6% before pumping to 46.7% after pumping, while PEDV RNA was detected in 91.6% of samples before and remained high at 84.4% after manure pumping (25). Thus, the ability of these viruses to survive in fecal samples and move from swine site to site through aerosolized manure particles should be accounted for in the bio-exclusion and bio-containment protocols in pig production systems.

Biosecurity protocols aim to minimize the introduction and spread of infectious pathogens within pig sites. Bio-exclusion measures are designed to prevent pathogens from entering a herd, including restricted farm access, sanitation protocols for personnel and equipment, and air filtration systems to mitigate airborne transmission of pathogens such as PRRSV and PEDV (26–28). Bio-containment measures, on the other hand, are intended to prevent the spread of pathogens from an infected herd to other farms, which includes strict handling and disposal of potentially contaminated materials such as manure, deadstock, and equipment. Therefore, manure management plays a critical role in biosecurity, as pathogens present in manure can become aerosolized during handling processes like pumping and spreading, posing a significant risk for both indirect transmission and environmental contamination. Effective biosecurity practices can significantly reduce the risk of airborne pathogens like PRRSV and PEDV traveling between sites, especially in areas where farms are in close proximity.

The unintended consequences of manure pumping and application to crop fields, including drops in barn temperature, agitation of pigs, and increased levels of various gasses, may lead to effective contact between pigs and pathogens deposited in the pit and carried over by contaminated equipment, increasing the risk of disease outbreaks. Additionally, the disposal of manure contaminated with pathogens (e.g., manure derived from diseased pig sites) in surrounding crop fields can likely increase disease pressure and outbreaks in previously pathogen-negative pig sites. Vilalta et al. (29) estimated the incidence risk ratio of PRRSV outbreaks within a 15- and 30-day window following manure pumping in 150 breeding herds. It was found that the incidence risk ratio was 9.55 (95% confidence interval [CI]: 6.59–13.84) for the 15-day window and 9.08 (95% CI: 6.81–13.49) for the 30-day window period after manure pumping (29, 30). There is no available data in the literature quantifying estimates of the association between manure pumping activities and disease outbreaks in wean-to-market sites. The objectives of this study were to measure the association of wean-to-market pig lots reporting PRRSV or PEDV outbreaks within 5 weeks after performing manure practices and to identify manure pumping and receiving-related risk factors associated with reporting PRRSV and PEDV outbreaks.

## 2 Materials and methods

### 2.1 Overview

**Figure 1:**
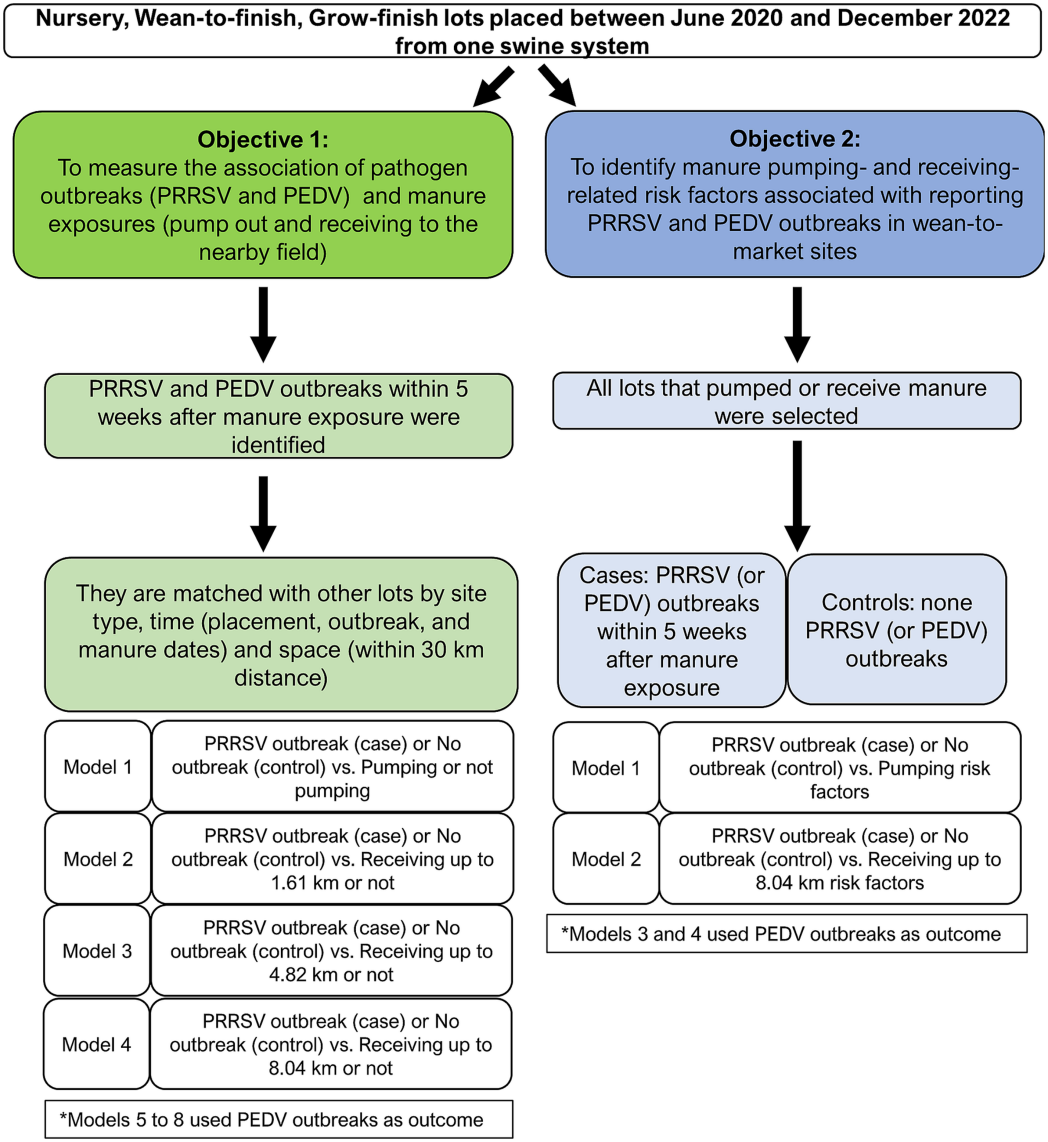
Overview of the study.

This was a retrospective observational study that included two objectives (Figure 1). The first objective was to estimate the association of wean-to-market pig lots reporting PRRSV or PEDV outbreaks within 5 weeks after performing two types of manure exposure (pumping out from the site or receiving the manure to the adjacent crop field of the site) using a matched case–control design. The population of interest was pig lots placed in nursery, wean-to-finish, and grow-finish sites between July 2020 and December 2022 in one pig production system in the United States Midwestern region. Cases were lots reporting PRRSV or PEDV outbreaks, while controls were lots that did not report a disease outbreak. For case–control matching purposes, all lots that broke with PRRSV or PEDV within 5 weeks post-pumping or -receiving manure were identified, with matching performed using temporal variables (pairing of lots placed within 4-week intervals of each other, week of PRRSV or PEDV outbreaks [when reported], week of manure pumping or receiving manure [when reported]), spatial (clusters created with a 30 km maximum pairwise distance among sites), and production site type (nursery, wean-to-finish, or grow-finish). The exposure events of pumping out manure or receiving manure to the adjacent crop field at different distances (1.61, 4.82, and 8.04 km radius) were considered the exposures in separate conditional logistic regression analyses (a total of eight models).

The second objective was to identify manure pumping- and receiving-related risk factors associated with reporting PRRSV and PEDV outbreaks in wean-to-market sites. The population of interest included lots that reported at least one manure pumping or receiving event. Herein, the cases were lots that reported PRRSV or PEDV outbreaks within 5 weeks post-pumping or receiving manure, while controls did not report any outbreak during their production period. The risk factors assessed in multivariable analyses were related to the characteristics of lots of pigs and equipment to store manure and apply manure to the crop fields within up to 8.04 km from the recipient site.

### 2.2 The association of wean-to-market lots reporting PRRSV or PEDV outbreaks within 5 weeks after performing two manure practices

#### 2.2.1 Data collection and management

A total of six datasets were retrieved for the analysis:

1) *Movement Dataset*: This included the lot identifier, wean-to-market site identifier, placement and closeout dates, and the total number of pigs placed.
2) *Daily Mortality Dataset*: This contained the lot identifier, wean-to-market site identifier, the number of dead pigs per day, and death dates.
3) *Outbreak Reports Dataset*: Managed by the swine system’s veterinary team, this dataset included PRRSV and PEDV outbreak reports based on clinical observations (such as increased mortality) from herd veterinarians, confirmed using diagnostic testing (PCR for RNA detection in tissues), disease diagnoses (associated gross and histopathological lesions), and sequencing (PRRSV open reading frame 5 [ORF5]) using well-validated assays implemented in one American Association of Veterinary Laboratory Diagnosticians (AAVLD)-accredited laboratory.
4) *Pathogen Monitoring Reports Dataset*: Retrieved from one AAVLD-accredited veterinary diagnostic laboratory, this dataset included a wean-to-market site identifier, confirmation of PRRSV or PEDV diagnoses (RNA detection in tissues and associated gross and histopathological lesions) by the lab’s diagnosticians and their dates. When available, PRRSV ORF5 sequencing was also retrieved.
5) *Pumping Management Dataset*: This dataset included the wean-to-market site identifier that was pumped, type of manure storage (deep pit under slats or external lagoon), type of manure transportation following pumping (tank hauled by truck or drag hoses), manure pumping dates, number of pumping events per site, the crop field identifier (the crop field that received the manure), amount of manure applied (in gallons), and the area covered with manure application (in acres). The swine system manages manure pumping operations at all its sites by hiring third-party contractors and defining pumping schedules, covering 100% of the pit pumping activities. However, the system does not have full control over manure spreading on adjacent crop fields of its sites. This means that some crop fields may receive manure from sites of different swine systems.
6) *Site-Specific Buffers Dataset*: This dataset was generated using satellite remote sensing connected to a georeferencing system (ArcGIS, ESRI 2011). It included 1.61 km (1 mile), 4.82 km (3 miles), and 8.04 km (5 miles) radius buffers surrounding sites that performed manure pumping events between July 2020 and December 2022. It also contained crop field identifiers surrounding the centered site at the specified distances.
7) *Sow Farm-derived disease status dataset:* To determine PRRSV statuses at the placement of lots, the dataset derived from Magalhães et al. (31) was used. This dataset included the wean-to-market lot identifier, corresponding sow farm(s) identifier, and PRRSV statuses of the lot. As described in Magalhães et al. (31), the epidemic status included the first 16 weeks after a PRRSV outbreak in the sow farm, with the presence of clinical signs compatible with PRRSV. The endemic status included week 17 after the outbreak until the herd was determined negative based on diagnostic testing. The negative status indicated the absence of nucleic acid detection by laboratory tests and clinical signs in the sow and piglet population.

The daily events were aggregated into weeks (ISO week date standard, ISO-8601), and thereby, the six datasets were connected using the pig lot identifiers (one per row). When the dataset did not include a pig lot identifier, combining a wean-to-market site identifier and dates of events was used to join datasets. Data management was done utilizing *dplyr* and *tidyverse* packages in R statistical software (R version 4.2.2).

#### 2.2.2 Outcome definition

In this study, cases were defined as lots reporting a PRRSV or PEDV outbreak. An outbreak was defined through the evidence of detection of these pathogens in tissue samples. The trigger for the collection of sample types for diagnosis was based on the herd veterinarian’s assessment of clinical history (clinical history of mortality and sick animals). Thus, it included all lots recorded in the *Outbreak Reports Dataset*. The swine system classifies sites based on biosecurity and health status as either ‘protected’ or ‘non-protected,’ with ‘protected’ sites requiring heightened surveillance, particularly those located near sow farms. Consequently, the *Outbreak Reports Dataset* was more likely to include “protected” sites. To address the potential underrepresentation of “non-protected” sites, the clinical reports in the *Pathogen Monitoring Reports Dataset* were also used to define cases. Those reports included pathogen detection in tissues with clinical history of mortality and sick animals. PRRSV sequencing (ORF5) was reported when available. Lots that included PRRSV status at placement as endemic or epidemic and reported an outbreak with associated ORF5 had the percent of diversity calculated to compare the strain of the sow farm and wean-to-market closeout. The Needleman–Wunsch global alignment algorithm was used for the alignment (32). Controls were defined as pig lots that did not report any pathogen detection during their production period.

#### 2.2.3 Exposure definition (manure pumping out and receiving manure in nearby crop field)

The pumping manure exposure was identified in lots that underwent at least one manure pumping event at the site where they were located. The receiving manure exposure variable referred to lots that received manure at least once in adjacent crop fields within 1.61 km, 4.82 km, or 8.04 km of the site. A pumping and receiving manure event referred to a continuous manure removal or application operation conducted at a single site (e.g., a barn or manure pit) or crop field over a defined period, typically completed within 1 day. Thus, those events included multiple truck visits depending on the size of the pit, earthen lagoon, and crop field.

To isolate the effect of receiving manure from pumping practices, the analysis excluded lots that were pumped and focused only on those receiving manure from other pumped sites. Furthermore, only protected sites were included, as the swine system manages 100% of manure pumping and receiving operations for these sites.

#### 2.2.4 Matched case–control description

The onset of shedding and clinical signs related to PRRSV and PEDV diseases typically occurs within 4 weeks post-infection in a pig population (13–15). Thus, the association between manure pumping and receiving was investigated in lots that reported an outbreak within 5 weeks following these events. This 5-week window between manure pumping and receiving events and outbreaks was chosen to allow for the PRRSV or PEDV exposure time and spread within the populations, plus an additional week to account for delays in outbreak investigation and reporting.

Lots were firstly matched by placement week; lots placed within a 4-week interval were grouped together. Thereafter, due to the wean-to-market sites of the system being geographically close, which increases the risk of disease transmission due to environmental conditions and the movement of animals, people, and equipment for manure handling, the sites were grouped in spatial clusters. Given that the reported distance from which PRRSV can be detected from the infectious source is 10 km (21, 22), and that pumping contractors from this dataset were reported to transport manure and equipment a median of 25 km within a 4-week interval (data not shown), clusters were defined using threshold-based spatial clustering with a 30 km pairwise threshold. A customized function based on the single-link cluster method in R was created to assign lot identifiers and their corresponding latitude and longitude locations to clusters based on a maximum allowable pairwise distance of 30 km (33). A random unassigned lot identifier started the process, and then all other lot identifiers within 30 km of this initial point (using the Haversine formula) were included to create the cluster. The process continued iteratively, forming clusters where each lot identifier was within 30 km of at least one other lot identifier in the same cluster.

The matching strategy involved aligning data temporally, i.e., with respect to placement, pumping, and outbreak dates, and spatially, i.e., by grouping sites within defined geographical clusters. The matching groups were: (1) all lots that had an outbreak within 5 weeks post-pumping, identified as “YES PUMP YES OUTBREAK” lots; (2) “NO PUMP NO OUTBREAK” lots were paired by placement week, cluster, and site type; (3) “NO PUMP YES OUTBREAK” lots were paired by the week of PRRSV or PEDV outbreak; and (4) “YES PUMP NO OUTBREAK” lots were paired by the week of pumping or receiving events. The matching considered the full production cycle of the studied lots. Lots that experienced a PRRSV or PEDV outbreak before the pumping event were excluded from the analysis. The same process was performed with the receiving manure exposure variable, i.e., receiving manure up to 1.61, 4.82, and 8.04 km.

#### 2.2.5 Statistical analyses

The unit of analysis was the pig lot. A conditional logistic regression was used to estimate the odds ratios of a PRRSV or PEDV outbreak (two separate analyses) and 95% confidence intervals (CIs) adjusted by site type (nursery, wean-to-finish, and grow-finish), and stratified by the placement week, pumping or receiving week, and geographic location (cluster with a 30 km radius) using the *survival* R package. Eight separate models were performed; e.g., PRRSV outbreaks were treated as the dependent variable with the following independent variables: site type (variable of adjustment, Models 1–8), pumping event (Model 1), receiving event up to 1.61 km (Model 2), receiving event up to 4.82 km (Model 3), and receiving event up to 8.04 km (Model 4). Models 5 to 8 included PEDV outbreak as the dependent variable and pumping and receiving up to 1.61 km, 4.82 km, and 8.04 km as independent variables. Models that included manure receiving independent variables (Models 2 to 4 and 6 to 8) only included sites classified as protected by the system because the site-specific buffers dataset only included protected sites. Model performance was assessed by evaluating residuals versus expected values using binned plots (*arm* R package) and likelihood tests (*p*-value < 0.50).

### 2.3 Identification of manure pumping- and receiving-related risk factors associated with reporting PRRSV and PEDV outbreaks in wean-to-market sites

#### 2.3.1 Data collection

All lots that included at least one event of manure pumping or receiving manure to the adjacent crop field from a different site up to 1.61, 4.82, or 8.04 km were retrieved from the master table created in Objective 1.

#### 2.3.2 Outcome definition

The cases were lots that reported PRRSV and PEDV outbreaks within 5 weeks after pumping or receiving manure from a different site. Controls did not report any PRRSV or PEDV outbreaks during their production period.

#### 2.3.3 Risk factors – manure pumping and receiving practices

The manure pumping and receiving risk factor variables are listed in Table 1.

**Table 1:** Manure pumping and receiving independent variables.

Variable type	Pumping out variables	Receiving variables
Categorical (categorized quantitative variables)	Herd size (<=5,000, 5,000-10,000, >10,000 pigs)	Herd size (<=5,000, 5,000-10,000, >10,000 pigs)
Categorical	Site type (wean-to-market, nursery, grow-finish)	Site type (wean-to-market, nursery, grow-finish)
Continuous (analyzed in specific categories)	Pumping week in relation weeks since placement (1–4 weeks, 5–11 weeks, 12–17 weeks, >17 weeks)	Spreading week in relation weeks since placement (1–4 weeks, 5–11 weeks, 12–17 weeks, >17 weeks)
Categorical	Ventilation type (natural or tunnel)	Ventilation type (natural or tunnel)
Categorical	Transportation method (tank hauled by trucks or drag hose)	Transportation method (tank hauled by trucks or drag hose)
Categorical	Facility storage type (earthen lagoon/concrete vats vs. underneath barn deep pits)	
Continuous (quantiles/categories)		Gallons applied
Continuous (quantiles/categories)		Size of recipient area (hectares)
Categorical	PRRSV outbreak in the previous lot placed in the site (yes, no)	Manure derived from lot of pigs with PRRSV outbreak in the previous 14 weeks of the pumping (yes, no)
Categorical		Distance of manure application from site (1.61 km, 4.82 km, or 8.04 km)
Continuous		Number of manure sources

Continuous variables related to manure pumping and receiving practices were categorized into quantiles (applied gallons and size of the recipient area in hectares) or in categories to enhance applicability to field conditions in swine production. For example, herd size was categorized as small (less than or equal to 5,000 pigs), medium (between 5,000 and less than or equal to 10,000 pigs), and large (more than 10,000 pigs). Weeks since placement when manure pumping or receiving was performed were categorized as follows: between the 1st and 4th week, between the 5th and 11th week, between the 12th and 17th week, and after the 17th week.

#### 2.3.4 Statistical analyses

The unit of analysis for all models in Objective 2 was the pig lot. Mixed-effects logistic regression was used to compare the odds of a PRRSV or PEDV outbreak (dependent variables in separate analyses) with manure pumping- and receiving-related practices (independent variables in separate analyses). Comparisons of outbreak odds were based on the first manure application for each lot, whether pumped or received. Only pig lots that received manure from a different site were included in the analysis. Independent variables were first tested with the dependent variables in a univariate manner adjusted by season and year of the placement week (covariates) and site identifier as a random effect. Variables with *p-value* ≤ 0.20 were then offered to the multivariate model. Multivariate models with variables of *p-value* ≤ 0.05 were retained in the final model for further interpretation. Multicollinearity was assessed in the final model through the variance inflation factor (VIF); if the VIF was higher than 2, a variable was removed due to multicollinearity. The models’ performance was assessed by evaluating residuals versus expected values for systematic trends or patterns and influential observations. All analyses were performed using R statistical software (version 4.2.2).

## 3 Results

### 3.1 Dataset overview

A total of 2,593 lots were placed across 599 wean-to-market sites between June 2020 and December 2022, including nursery lots (134 lots, 5.2%), wean-to-market lots (2,113, 81.5%), and grow-finish lots (345 lots, 13.3%). Protected sites (*n* = 56 sites in total) housed a total of 264 lots of pigs (representing 10.2% of 2,593 lots) during the study period, of which 26.1% (69 lots) were nursery pigs, 57.2% (151 lots) were wean-to-finish, and 16.7% (44 lots) were grow-finish.

From the 2,593 lots, 1,370 (52.8%) lots had at least one pumping event, involving a total of 101 third-party contractors. Of those, 85.0% (1,165 sites) had only one pumping event, 13.0% (179) had two pumping events, 1.5% (20) had three pumping events, 0.49% (5) had four pumping events, and 0.01% (1) had six pumping events. Among sites with one pumping event, 46 (3.9%) were nurseries, 983 (84.4%) were wean-to-finish, and 136 (11.7%) were grow-finish. For sites with two pumping events, there were 7 (3.9%) nurseries, 148 (82.7%) were wean-to-finish, and 24 (13.4%) were grow-finish. Sites with three pumping events included one (5.0%) nursery, 14 (70.0%) were wean-to-finish, and five (25.0%) were grow-finish. Sites with four pumping events included one nursery and four wean-to-finish. The site with six pumping events was a wean-to-finish.

Receiving manure to adjacent site crop fields only included protected sites (*n* = 56) and their lots (264 lots). Of those, 198 lots (75%) received manure up to 8.04 km at least once during the study period, of which 52 lots (26.3%) were nursery sites, 104 (52.5%) were wean-to-finish, and 42 (21.2%) were grow-finish. A total of 41 lots (20.7%) experienced one receiving manure event, while 48 lots (24.2%) had two events. Three events were recorded for 32 lots (16.2%), and four events for 19 lots (9.6%). Additionally, 17 lots (8.6%) experienced five receiving manure events, 12 lots (6.1%) had six events, and 19 lots (9.6%) had seven events. Seven lots (3.5%) experienced eight events, one lot (0.5%) had nine events, and two lots (1.0%) experienced 14 receiving manure events.

From the 2,593 lots, 380 reported a PRRSV outbreak. Of those, 208 PRRSV outbreaks (54.7%) included RNA detection and ORF sequencing, while the others included RNA detection in tissues with a disease diagnosis of PRRSV. Of the 208 sequenced PRRSV outbreaks, 129 (62.0% of 208) were associated with L1C.5 1-4-4, followed by 26 (12.5%) L1A 1-7-4.

Additionally, of the 380 PRRSV outbreaks, 40 sites (10.5%) were nursery, 288 sites (75.8%) were wean-to-finish, and 52 sites (13.7%) were finishing. A total of 103 lots reported a PEDV outbreak; 79 sites (76.7%) were wean-to-finish, and 24 sites (23.3%) were grow-finish.

As shown in Table 2, 37.4% of all manure pumping and receiving events in this study occurred in Fall 2021, followed by Fall 2022 (27.9%) and Fall 2020 (15.3%). Likewise, the majority of the PRRSV outbreaks were in Fall 2021 (20.3%), followed by Fall 2022 (16.0%) and Spring 2021 (14.5%). In contrast, the majority of PEDV outbreaks occurred in Winter 2021 (35.6%), followed by Winter and Spring 2022 (both 17.8%).

**Table 2:** Seasonal distribution of manure pumping, receiving events, and outbreaks of porcine reproductive and respiratory syndrome virus (PRRSV) and porcine epidemic diarrhea virus (PEDV) between June 2020 and December 2022 in wean-to-market lots from one swine system.

Season/year	Pumping and receiving events (%)	PRRSV outbreaks (%)	PEDV outbreaks (%)
Summer 2020	0.1%	0.5%	0%
Fall 2020	15.3%	5.3%	0%
Winter 2020	0.7%	3.9%	2.2%
Spring 2021	6.7%	14.5%	0%
Summer 2021	0.1%	7.9%	13.3%
Fall 2021	37.4%	20.3%	8.9%
Winter 2021	3.4%	8.4%	35.6%
Spring 2022	8.0%	6.6%	17.8%
Summer 2022	0.4%	8.4%	2.2%
Fall 2022	27.9%	16.0%	2.2%
Winter 2022	0.2%	8.2%	17.8%
Total % (number of pig lots)	100% (1,370)	100% (380)	100% (103)

### 3.2 The association of wean-to-market lots reporting PRRSV or PEDV outbreaks within 5 weeks after performing two manure practices

#### 3.2.1 Association of PRRSV outbreaks following manure pumping and receiving events

Of all 380 PRRSV lot outbreaks, 115 occurred before any manure pumping activity and were therefore excluded from the analysis. Using a case–control matching design, 80 lot outbreaks were identified as occurring within 5 weeks following a manure pumping event. These 80 lots were matched with lots in the same geographical clusters and with aligned placement, pumping, and outbreak dates. The matching selection included a total of 58 clusters, totaling 307 lots included in the analysis. Of those, 160 had at least one pumping event (52.2%), and 116 (37.8%) reported a PRRSV outbreak. The case–control ratio was 2 controls for every 1 case. Of the 307 lots included in the analysis, 87 (28.3%) were classified as negative at placement and were otherwise classified as endemic at placement. Of the 116 lots that reported a PRRSV outbreak, 55 (48.2%) were considered negative at placement. The other 61 lots reporting a PRRSV outbreak were classified as endemic; the ORF5 of 22 lots had an ORF5 sequence with temporal alignment between the sow farm source and wean-to-market. A total of four of the 22 ORF5 sequences obtained less than 2% homology (range: 98.7 to 99.8%).

For the receiving analysis, only pig lots from protected sites receiving manure from a different protected site were included. For receiving up to 1.61 km, a total of 64 lots were included; of those, 30 received manure up to 1.61 km and 32 had outbreaks (i.e., 18 had outbreaks within 5 weeks from the receiving manure up to 1.61 km). Of the 64 lots, 41 (64.1%) were classified as negative at placement, with 24 (75.0%) of the 32 reporting the PRRSV outbreak classified as negative.

For receiving up to 4.02 km, a total of 91 lots were included; 62 received manure up to 4.02 km, and 39 had an outbreak (i.e., 29 had an outbreak within 5 weeks from receiving manure up to 4.02 km). Of the 91 lots, 70 (76.9%) were classified as negative at placement, with all 39 lots reporting the PRRSV outbreak classified as negative.

For receiving up to 8.04 km, a total of 96 lots were included; 74 received manure up to 8.04 km, and 43 had an outbreak (i.e., 36 had an outbreak within 5 weeks from receiving manure up to 8.04 km). Of the 96 lots, 58 (60.4%) were classified as negative at placement, with 34 (79.1%) of the 43 reporting the PRRSV outbreak classified as negative.

Manure pumping and receiving events were both associated with higher odds of PRRSV outbreak (Table 3). The odds of having a PRRSV outbreak within 5 weeks after pumping were 3.38 (95% CI 1.86–6.11, *p*-value < 0.001) times higher than in lots that were not pumped. Likewise, the odds of PRRSV outbreaks within 5 weeks after receiving manure up to 1.61 and 4.02 km distance were 4.09 and 3.05 times higher (*p*-value < 0.05), respectively, than in those not receiving manure.

**Table 3:** Association between porcine reproductive and respiratory syndrome virus (PRRSV) outbreak following manure pumping and receiving events in the previous 5 weeks using conditional logistic regression stratified by placement week, pumping or receiving manure week, geographic location, and site type (nursery, wean-to-finish, or grow-finish).

Model	Manure events	Number of lots	Odds ratio	95% CI	*P*-value
1	Not pumped	147	1	–	–
	Pumped within the previous 5 weeks	160	3.38	1.86–6.11	<0.001
2	Didn’t receive manure	34	1	–	–
	Receiving manure at up to 1.61 km (1 mile) within the previous 5 weeks	30	4.09	1.05–16.00	0.043
3	Didn’t receive manure	29	1	–	–
	Receiving manure at up to 4.82 km (3 miles) within the previous 5 weeks	62	3.05	1.12–8.27	0.028
4	Didn’t receive manure	22	1	–	–
	Receiving manure up to 8.04 km (5 miles) within the previous 5 weeks	74	2.20	0.71–6.89	0.168

#### 3.2.2 Association of PEDV outbreaks following manure pumping and spreading events

Of the 103 PEDV outbreaks, 30 lots had a pumping event; of those, only 4 outbreaks occurred within 5 weeks after the pumping week. Indeed, of the 103 PEDV outbreaks, 40% occurred in the first week of placement, 27% in the second week, and 13% in the third week (all before the pumping week), and then were removed from the analysis. One pig lot from the 103 case lots reported at least one manure receiving event. Thus, no associations between PEDV outbreak and manure pumping or receiving were detected in this dataset (*p*-value > 0.05). Therefore, no analyses comparing the odds of PEDV outbreak across different manure pumping and receiving practices were performed.

### 3.3 Identification of manure pumping- and spreading risk factors associated with reporting PRRSV and PEDV outbreaks in wean-to-market sites

#### 3.3.1 Comparison of odds of PRRSV outbreaks across manure pumping practices

A total of 80 lots that reported a PRRSV outbreak within 5 weeks after a manure pumping event (cases), along with 1,159 lots that underwent manure pumping but did not report any PRRSV outbreak during their production cycle (controls), were included in the analysis. Therefore, lots that had a PRRSV outbreak outside of the 5-week window and PEDV outbreaks were not included. In total, 1,239 pig lots across 564 sites with at least one recorded manure pumping event (median = 1.5 events per lot; range: 1–6) were used to evaluate the association between PRRSV outbreaks and pumping-related practices.

The univariate analysis (Table 4) revealed that pumping sites where there was a PRRSV outbreak in the previous lot placed at the site were associated with 3.52 times higher odds of having a PRRSV outbreak compared to lots that did not have a PRRSV outbreak in the previous lot (*p*-value = 0.001). Additionally, nursery lots were associated with 5.76 times higher PRRSV outbreak odds than finishing lots (*p*-value = 0.008). While sites that included deep pits rather than lagoons as storage and transported the manure from the pit to the crop field using tanks hauled by trucks rather than drag hoses were associated with lower odds of PRRSV outbreaks, 0.35 and 0.51, respectively.

**Table 4:** Association between porcine reproductive and respiratory syndrome virus (PRRSV) outbreak and manure pumping-related risk factors (univariate mixed effect logistic regression model adjusted by season and year [covariates] and site [random effect]).

Variables	Levels	Number of pig lots	Odds ratio	95% CI	*P*-value
PRRSV outbreak in the previous lot placed in the site	No	1,173	1		
	Yes	66	3.52	1.55–7.97	**0.003**
Ventilation	Natural	1,062	1		
	Tunnel	106	1.09	0.17–7.08	0.931
Site type	Finish	144	1		
	Nursery	53	5.76	1.36–24.37	**0.017**
	Wean-to-finish	1,042	1.16	0.46–2.92	0.751
Manure storage type	Lagoon	54	1		
	Deep pit	786	0.35	0.17–0.74	**0.006**
Manure transport method	Hose	268	1		
	Tank	971	0.51	0.27–0.95	**0.035**
Site size	Less than 5,000 pigs	478	1		
	Between 5,000 and 10,000 pigs	506	4.03	2.00–8.14	**<0.001**
	More than 10,000 pigs	255	6.75	3.24–14.06	**<0.001**
Pumping week in relation to the age of pigs	After 17th week	301	1		
	Between 12th and 17th week	306	3.17	0.95–10.56	0.060
	Between 5th and 11th week	376	3.42	1.11–10.54	**0.032**
	Between 1st and 4th week	256	5.64	1.76–18.08	**0.004**

Values presented in bold denote statistically significant differences at the 5% significance level (*p* < 0.05).

As the number of pigs in the lot increased, the odds of a PRRSV outbreak also increased; e.g., herds of medium (5,000 to 10,000 pigs) and large (more than 10,000 pigs) sizes were associated with higher odds of a PRRSV outbreak than relatively small herds (less than 5,000 pigs) (*p*-value < 0.001). In contrast, the odds of a PRRSV outbreak were higher by 5.64 times when pumping occurred before the 4th week of placement age compared to after the 17th week (*p*-value < 0.01). Variables such as PRRSV outbreak in the previous lot placed at the site, site type, storage type, transportation method, site size, manure storage type, and pumping event week in relation to the placement age in weeks were offered to the multivariate model (*p*-value ≤ 0.20).

The occurrence of a PRRSV outbreak in the previous lot placed at the site, site type, site size, and the age in weeks at the pumping event in relation to the placement age were retained in the final multivariable model (Table 5, *p*-value ≤ 0.05).

**Table 5:** Final multivariable mixed-effects logistic regression model adjusted by season and year (covariates), and site (random effect) of the association between porcine reproductive and respiratory syndrome virus (PRRSV) outbreak and manure pumping-related risk factors.

Variables	Levels	Number of pig lots	Odds ratio	95% CI	*P*-value
PRRSV outbreak in the previous lot placed in the site	No	1,173	1		
	Yes	66	3.00	1.47–6.14	**0.003**
Site type	Finish	144	1		
	Nursery	53	0.51	0.13–1.97	0.327
	Wean-to-finish	1,042	0.33	0.12–0.92	**0.033**
Site size	Less than 5,000 pigs	478	1		
	Between 5,000 and 10,000 pigs	506	4.49	2.15–9.37	**<0.001**
	More than 10,000 pigs	255	6.60	3.00–14.52	**<0.001**
Pumping week in relation to the age of pigs	After 17th week	301	1		
	Between 12th and 17^th^ week	306	3.76	1.22–11.57	**0.021**
	Between 5th and 11th week	376	4.12	1.32–12.92	**0.015**
	Between 1st and 4th week	256	6.13	1.81–20.72	**0.004**

Values presented in bold denote statistically significant differences at the 5% significance level (*p* < 0.05).

By holding all variables constant, lots that were pumped in a site that included a lot with a reported PRRSV outbreak were associated with 3.0 times higher PRRSV outbreak odds compared to not having a reported PRRSV outbreak. The odds of outbreaks were higher by 6.13 times in lots that were pumped between the 1st and 4th weeks of placement age. Herds of medium (5,000 to 10,000 pigs) and large (more than 10,000 pigs) sizes were associated with 4.49 and 6.60 times higher PRRSV outbreak odds compared to smaller sizes (less than 5,000 pigs).

#### 3.3.2 Comparison of odds of PRRSV outbreaks across manure spreading practices

A total of 123 pig lots (spreading events per lot median = 6, minimum = 1, maximum = 14) spreading manure in the adjacent crop fields (from 1.61 km to 8.04 km from the site) from a different site that was pumped were used to compare the odds of PRRSV outbreak and manure spreading-related practices. Of those, 41 lots reported PRRSV outbreaks within 4 weeks after spreading manure at 1.61, 4.62, and 8.04 km. Results from the univariate analysis are shown in Table 6.

**Table 6:** Association between porcine reproductive and respiratory syndrome virus (PRRSV) outbreak and manure receiving-related risk factors (univariate mixed effect logistic regression model adjusted by season and year [covariates] and site [random effect]).

Variables	Levels	Number pig of lots	Odds ratio	95% CI	*P-*value
Manure derived from lot of pigs with PRRSV outbreak in the previous 14 weeks of the pumping	No PRRSV outbreak	69	1	–	–
	PRRSV outbreak	57	1.37	0.57, 3.29	0.47
Ventilation	Natural	114	Not done (no variability)		
	Tunnel	1			
Site type	Finish	29	1	–	–
	Nursery	22	1.75	0.48, 6.59	0.39
	Wean-to-finish	75	0.49	0.18, 1.35	0.16
Number of manure sources	1	92	1	–	–
	2 or 3	34	2.09	0.88, 5.01	0.09
Manure transport method	Hose	23	1	–	–
	Tank	40	1.00	0.24, 4.68	0.99
Site size	Less than 5,000 pigs	37	1	–	–
	Between 5,000 and 10,000 pigs	48	1.65	0.54, 5.39	0.38
	More than 10,000 pigs	31	9.28	2.98, 32.48	<0.01
Spreading week in relation to the age of pigs	After 17th week	25	1	–	–
	Between 12th and 17th week	22	1.61	0.55, 7.47	0.47
	Between 5th and 11th week	54	2.48	0.85, 7.84	0.10
	Between 1st and 4th week	25	1.99	0.43, 6.13	0.30
Total acres covered in the first manure application	22–83	32	1	–	–
	84–144	32	1.49	0.45, 5.13	0.51
	145–277	31	1.49	0.40, 5.82	0.55
	278–1,423	31	2.28	0.65, 8.53	0.20
Total applied gallons in the first manure application	83,354–532,588	32	1	–	–
	549,174–902,812	31	3.10	0.91, 11.59	0.08
	929,952–1,745,786	33	2.75	0.78, 10.45	0.12
	1,772,305–4,741,116	30	3.99	1.12, 14.64	0.03
Distance from the site where manure was applied	4.82 < x ≤ 8.04 km	74	1	–	–
	1.61 < x ≤ 4.82 km	53	0.60	0.22, 1.56	0.30
	≤1.6 km	15	6.44	1.81, 27.70	<0.01

By holding all variables constant, the multivariate analyses showed that herds of large sizes (more than 10,000 pigs) were associated with 11.47 (*p*-value < 0.01, Table 5) times higher odds of PRRSV outbreak than small herds (less than 5,000 pigs), and sites where manure was spread within a 1.6 km distance from the site were significantly associated with 11.03 (*p*-value < 0.01, Table 7) times higher PRRSV outbreak odds than those between 4.82 and 8.04 km distances.

**Table 7:** Final multivariable mixed-effects logistic regression model adjusted by season and year (covariates), and site (random effect) of the association between porcine reproductive and respiratory syndrome virus (PRRSV) outbreak and manure receiving-related risk factors.

Variables	Levels	Odds ratio	95% CI		*P*-value
Site size	Less than 5,000 pigs	1	–		–
	Between 5,000 and 10,000 pigs	1.43	0.41	5.24	0.64
	More than 10,000 pigs	11.47	3.43	44.52	<0.01
Distance from the site where manure was applied	4.82 < x ≤ 8.04 km	1	–		–
	1.61 < x ≤ 4.82 km	0.70	0.23	2.73	0.53
	≤ 1.6. km	11.03	2.73	53.43	<0.01

## 4 Discussion

This study identified significant associations between manure management practices and PRRSV outbreaks. Pig lots that experienced pumping within the previous 5 weeks had 3.38 times higher odds of a PRRSV outbreak (Table 3), indicating a substantial effect of this practice on disease occurrence. Additionally, the studied closeout lots were mostly classified as negative at placement, supporting the hypothesis that manure pumping and receiving increased the risk of lateral outbreaks. In contrast, PRRSV outbreaks in lots classified as endemic following manure pumping may be associated with the exacerbation of ongoing infections caused by these activities.

The multivariate model of pumping-related risk factors revealed that pumping sites with a PRRSV outbreak in the previous lot were associated with 3.38 times higher odds of a PRRSV outbreak in the subsequent lot (Table 5). These aligned findings suggested that residual contamination or lingering viral presence at pumped sites remained infectious and viable in the manure, and that pumping and agitation in the deep pit or attached earthen lagoons may re-circulate the virus within the newly placed pig population (16, 19, 34).

Manure application created opportunities for virus dissemination through aerosols, overspill, and contamination of equipment. Specifically, increasing odds of PRRSV outbreaks in sites receiving manure to adjacent crop fields up to 4.82 km (3 miles) from the site housing the pigs was also statistically significant (Table 3). This risk appeared to decrease with distance, as indicated by non-significant and lower odds ratios of 2.20 at 8.04 km (5 miles). Both pumping and receiving events highlight the importance of proximity and timing in PRRSV transmission risk. Pumping events, which typically occur once or twice per lot, can result in concentrated exposure, while manure receiving events, particularly when frequent and involving large volumes, may present cumulative risks. These findings emphasized the need for targeted biosecurity measures, including stricter controls around pumping activities and careful management and disinfection of manure application equipment to minimize exposure risks.

Additionally, increased weekly mortality rates, one of the first clinical observations in wean-to-market pigs affected by PRRSV infections (9), could be observed within the first 2 weeks after the pumping event (data not shown). Thus, these results showed that the effect of manure pumping regarding the PRRSV outbreak could be observed within 5 weeks after the event, and veterinarians and producers can decide to use preventive measures (such as preventive medication to decrease the impact of infection by secondary pathogens and vaccination) before pumping events to mitigate the unintentional consequences of pumping events in the wean-to-market pig population.

Most PRRSV outbreaks were reported during the spring and fall seasons, with the highest frequency in spring 2021, likely associated with the PRRSV lineage 1C.5 (formerly known as RFLP 1-4-4 Lineage 1C variant) (35, 36). The seasonal trends observed could be attributed to environmental conditions that favor the persistence and transmission of the virus, such as cooler temperatures and higher humidity during these periods. Additionally, the timing of manure handling practices, predominantly in the fall, might exacerbate the risk of PRRSV outbreaks due to the confluence of these factors.

Conversely, no significant association was found between manure practices and PEDV outbreaks in the dataset analyzed. This finding can be explained by an insufficient number of lot cases that reported pumping events; for example, 30 of the 103 lot cases reported at least one pumping event, and only 4 lots reported a PEDV outbreak within 5 weeks following the pumping event. Tun et al. (37) showed that PEDV was detected up to 9 months in earthen lagoons and that the PEDV viral load in the top layer of lagoons was low and primarily non-infective, suggesting that UV light and sunlight could diminish the replicability and infectivity of the virus. A prospective study performed in the same swine system as this study, but including 86 wean-to-market barns from 36 sites that pumped in 2023, collected oral fluid and manure pig samples tested by PCR for PRRSV and PEDV detection, and showed that only two of the 74 collected oral fluid samples tested PEDV-positive post-pumping (25). These studies suggested that while manure management was an important factor in PRRSV transmission, no statistically significant association was observed for PEDV outbreaks. This may reflect differences in transmission dynamics between the two viruses, although the limited number of PEDV cases may have reduced the power to detect such associations. In fact, 80% of reported PEDV outbreaks occurred within the first 3 weeks post-placement, which is indicative of disruptions more likely due to transportation from the sow farm or nursery to the grow-finishers.

Logistics of manure pumping and receiving significantly impacted the odds of PRRSV outbreaks within 5 weeks after manure management events. The univariate analyses of pumping risk factors showed that lots where manure was hauled by truck were associated with 45% lower odds of PRRSV outbreaks compared to lots where drag hose systems were used. This aligned with observations from field experts. There is a critical difference in biosecurity practices and operational features between the drag hose systems and tanks hauled by trucks. Truck applicators typically wash out vehicles, pumps, and hoses between sites, reducing the potential for cross-contamination. That is, truck-mounted manure hauling tanks can be cleaned using heat-based methods such as baking between sites. In contrast, drag hose systems, which involve 1.62–3.22 km (1–2 miles) long hoses and operate under high pressure, typically serve one single farm; however, due to the design, they can present challenges for thorough cleaning due to the large volume of residual manure and limited flushing efficiency, which can pose potential biosecurity risks from pathogen carryover. Nonetheless, drag hose systems can have practical advantages, such as cost benefits (manure is 95% water) and timely application to nearby crop fields. In the current study, we also observed that proximity played a significant role in PRRSV transmission. For instance, lots receiving manure within 1.62 km (1 mile) had 11.03 times higher odds of a PRRSV outbreak compared to those receiving manure at 8.04 km (5 miles), consistent with the hypothesis that shorter distances facilitate greater exposure to viral pathogens. This finding supports the need to evaluate trade-offs between operational efficiency and biosecurity risks when selecting manure application methods.

This study revealed an unexpected association between manure storage type and PRRSV outbreaks. Deep pit barns, which house pigs directly above the stored manure, were associated with significantly lower odds of PRRSV outbreaks compared to lagoon or vat systems (OR = 0.35, Table 4). This finding is counterintuitive to the hypothesis that deep pit systems, where pigs remain nearest to the manure during pumping, would pose a greater risk due to potential aerosolization or direct contact with viral particles. A potential explanation for this discrepancy lies in the site types included in the study. Nurseries, the populations with higher odds of PRRSV breaks as highlighted in the multivariable models, included only lagoon or concrete vat storage. This imbalance may have influenced the results, as deep pit barns were predominantly used in wean-to-finish and grow-finish operations, which may inherently differ in outbreak risk.

Herd characteristics also played a role in PRRS outbreaks following pumping. The size of the herd significantly impacted the odds of a PRRSV outbreak following pumping and receiving events, with larger herds (more than 10,000 pigs) having up to 6.75 times higher odds of an outbreak compared to smaller herds (less than 5,000 pigs) following pumping events. Given that large sites represented nursery pigs (a variable that also resulted in a statistical association), this result revealed that age was also a contributing factor to PRRSV outbreaks following pumping events. Moreover, the timing of manure application relative to the placement of pigs was critical. Lots that pumped manure later in the production cycle (after the 17th week) had significantly lower odds of PRRSV outbreaks. These results suggested that delaying manure handling until later stages of production may be an effective strategy to reduce the risk of PRRSV lateral outbreaks, possibly because younger pigs are more susceptible to infection.

Another critical factor contributing to the higher odds of PRRSV outbreaks associated with manure pumping and receiving events is the logistical complexity and the involvement of multiple companies in the process; e.g., 101 third-party pumping contractors were involved in manure pumping and receiving activities in the studied swine system. Manure pumping is typically managed by a single contractor directly involved with the site, allowing for greater control over biosecurity measures. In contrast, due to the size of crop fields, manure receiving was often performed by more than one different third-party contractor applying manure to multiple crop fields surrounding a single recipient site. This introduced variability in the execution of biosecurity protocols and multiple visits to the same site, increasing the number of events for PRRSV transmission.

Different third-party pumping contractors alongside the surrounding swine companies may have varying levels of adherence to biosecurity practices, leading to inconsistent application of measures designed to prevent the spread of PRRSV. The involvement of multiple contractors also increases the number of personnel, equipment, and vehicles moving between farms, which can facilitate the spread of the virus if proper decontamination procedures are not rigorously followed. In addition, manure pumping and spreading activities conducted in regions with high swine farm density often involve multiple farms from different production systems operating simultaneously and may share third-party pumping contractors. As a result, there is limited control over potential exposure to manure applications performed by other swine companies or untracked sites in the surrounding area. A study on *Actinobacillus pleuropneumoniae* serotype 15 outbreaks in grow-finish pigs across nine production systems found that third-party rendering contractors were a significant biosecurity risk to the spread of the bacteria within a 40 km radius from the index case (38). This could be due to their potential to work with multiple producers, some of whom may have unknown disease statuses, thereby increasing the likelihood of disease transmission and biosecurity breaches.

Although ventilation status during manure pumping events was not recorded in this study, increasing airflow, i.e., typically through the opening of sidewall curtains, is a common and recommended practice to improve internal air quality (39). Monitoring ventilation conditions is recommended during pumping activities, as proper ventilation may help mitigate the accumulation of airborne contaminants such as dust, ammonia, carbon dioxide, and microbial particles. These factors have been associated with increased susceptibility to respiratory diseases and may influence pig health during and after manure handling activities (40).

While this study included a large sample size, certain limitations should be acknowledged. One of the key limitations was the underestimation of PRRSV outbreaks in the control sites (sites with no reports of pathogen detection in tissue samples). The data relied on reports from herd veterinarians and their pursuit of diagnostic testing, which might not occur uniformly across all wean-to-market herds. That is, subclinical outbreaks (with milder clinical signs and changes in mortality rates), particularly in non-protected sites, were overlooked. Insufficient observations of PRRSV ORF5 sequence alignment between pumped manure sender sites and receiving sites limited the confirmation of potential associations.

Another key limitation was that the data on manure receiving events was limited to protected sites due to the lack of complete information about the other named non-protected sites. Thus, it was not possible to capture the full range of conditions under which manure spreading to the crop fields occurred, potentially limiting the internal validation of results. Lastly, this study was based on retrospective data, which can pose challenges related to data completeness and consistency. To mitigate this, we selected proper controls and adjusted for potential confounders. Despite the inherent challenges of retrospective data, our findings consistently showed significant associations, suggesting that manure pumping and receiving were risk factors for PRRSV outbreaks in wean-to-market populations.

## 5 Conclusion

This study identified a significant association between PRRSV outbreaks and manure pumping, as well as receiving manure applied onto crop fields from different pumped site sources at distances of ≤1.61 km and up to 4.82 km. Given that manure pumping due to limited storage space and application to fertilize crop fields are necessary, this study estimated which risk factors can potentially be adjusted and considered to prevent PRRSV outbreaks in wean-to-market sites. Risk factors associated with the odds of PRRSV outbreaks included herd size (medium to large herds and nurseries), week of pumping (interval of 1st – 4th week post-placement), and prior PRRSV-positive lots in the pumped site. PRRSV outbreaks following manure exposure from adjacent fields were associated with herd size (large herds) and closer distances <1.61 km. These insights can guide producers and veterinarians in implementing preventive measures to reduce PRRSV transmission following manure handling procedures. No statistically significant association was observed between this practice and PEDV outbreaks. However, the limited number of PEDV-positive lots observed in the study’s retrospective data may have reduced the power to detect a true effect.

## Data Availability

The datasets presented in this article are not readily available because the data used in this study will not be shared publicly. Access to the data is restricted to authorized individuals and institutions in compliance with the agreed-upon terms. Requests to access the datasets should be directed to gustavos@iastate.edu.
